# The study of antecedent clinical manifestations of hypertensive heart disease in cohort of hypertension

**DOI:** 10.18632/aging.204510

**Published:** 2023-02-19

**Authors:** Qian Li, Na Li, Xiao Liang, Yanjie Liu, Li Chen, Huimin Lao, Sheng Wei, Jun Xiao, Xiaoqiang Qi

**Affiliations:** 1 The American Academy of Tradition Chinese Medicine Inc. Roseville, MN 55113, USA; 2 Qingdao Fifth People’s Hospital, Qingdao 266002, Shandong, China; 3 Saint Mary’s University of Minnesota, Minneapolis, MN 55404, USA; 4 Department of Neck-Shoulder-Lumbocrural Pain Treatment, Yantai Hospital of Traditional Chinese Medicine, Yantai 264013, Shandong, China; 5 Department of Science and Education, Shandong Provincial Third Hospital, Jinan 250031, Shandong, China; 6 Shandong University of Traditional Chinese Medicine Affiliated Hospital, Jinan 250011, Shandong, China; 7 Experimental Center, Shandong University of Traditional Chinese Medicine, Jinan 250355, Shandong, China; 8 Key Laboratory of Traditional Chinese Medicine Classical Theory, Ministry of Education, Shandong University of Traditional Chinese Medicine, Jinan 250355, Shandong, China; 9 The Macrohard Institute of Health, Roseville, MI 48066, USA; 10 University of Missouri-Columbia, Columbia, MO 65212, USA

**Keywords:** hypertensive heart disease (HHD), hypertension, palpitation, hypertension symptoms

## Abstract

Hypertensive heart disease presents increasing morbidity and mortality worldwide, however, the data about its epidemics and its specific symptoms in hypertension patients is scarce. To assess the frequency and correlated symptoms of hypertensive heart disease, 800 hypertension patients were randomly recruited for this study per the guidelines of the American College of Cardiology. The diagnosis of heart disease and its typical symptoms (palpitation and angina) were analyzed for the frequency of hypertensive heart disease in hypertension cohort. Cross-tabulation analysis was used to study the correlation between psychiatric indexes (annoy, amnesia, irritableness, depression, anxiety, and fear) and palpitation, the correlation between physical disorders (backache, lumbar debility, and numbness of limbs) and palpitation, and the correlation between symptoms (dizziness, daze, headache, and tinnitus) and palpitation presented in hypertensive patients. It was found that around half of patients suffered hypertensive heart disease, which correlated to certain physical and mental symptoms. Significant correlation exists between palpitation and annoy / amnesia. Significant correlation exists between palpitation and backache / lumbar debility / numbness of limbs; and significant correlation exists between palpitation and dizziness / daze / headache / tinnitus. These results provide clinical insights into the modifiable antecedent clinical conditions which are risk factors for hypertensive heart disease in elderly and will help improve early management of this disease.

## INTRODUCTION

Complications caused by hypertension have been a prevalent public health issue, further, the cardiac complications are the main cause of morbidity and mortality in the patients with high blood pressure [1, 2]. The first definition of hypertensive heart disease (HHD) was proposed in 1979 by the New York Heart Association, which defined HHD as an anatomo-functional alteration characterized by left ventricular hypertrophy (LVH) and cardiac failure in patients with systemic hypertension [3]. In 1992, Frohlich et al. defined HHD as the response of myocardium to the afterload imposed by increased blood pressure that leads to LVH [4]. However, the comprehensive definition of HHD could be a complex and variable syndrome including clinical manifestations derived from LVH, myocardial ischemia and rhythm abnormalities (arrhythmia), which are obviously related to each other and all of which can be resulted from the effects of high blood pressure on the heart [5–7].

Age may be the paramount risk factor for HHD, but superimposed on age are modifiable antecedent clinical conditions, the first of which is hypertension [8–12]. Therefore, preventing hypertension is an effective preventive measure to decrease HHD prevalence in elderly. Indeed, hypertension has already been a major public health issue with a remarkable morbidity, and the prevalence of hypertension has reached alarming proportions worldwide with rapid economic development and lifestyle changes [13]. It was estimated that 30% of population worldwide would suffer hypertension within next decade [14, 15]. Hypertension has been known as a strong risk factor for heart disease, potentially lethal ventricular arrhythmias and sudden cardiac death occurred more common in hypertensive patients than others [16]. Existing studies on hypertension symptoms focused on the relationship between symptoms and quality of life [17], the associations of symptom relief and treatments [18, 19], and the symptom differentiations of normotensives, borderline hypertensives and hypertensives [20]. To our knowledge, no studies have investigated the correlations among different symptoms of hypertension and the correlations between each symptom and HHD. Such study is not only interesting, but also of great clinical significance. A variety of symptoms may be directly or indirectly related to hypertension, such as dizziness, nervousness, sweating, sleep disorder, facial flushing, and blood spots in the eyes, etc. Some of those symptoms might not only be caused by high blood pressure but also caused by hypertension involved heart diseases. The MAYO CLINIC has outlined some symptoms from HHD. Chest pain, chest tightness and angina could be main clinical manifestation of heart diseases originated from hypertension, also shortness of breath, dizziness, pain in the neck, pain in the back, pain or numbness in the limbs are often seen in patients with HHD. However, persistent cough, swelling in legs, hands, ankles or feet more likely result from heart diseases originated from cardiomyopathy or heart infection and heart defects. Exploring the correlations of distinct symptoms and HHD could be interesting and meaningful. In addition, although higher than normal blood pressures can be objectively measured, symptom observation plays an indispensable role because eventually, we treat the symptoms as well as the underlying conditions but not the numbers. Therefore, statistically studying the prevalence of each symptom and the correlations among each symptom based on a large population will provide important insight into cost-effective long-term hypertension management and HHD control.

Furthermore, although HHD is the number one cause of death associated with high blood pressure [21], original clinical studies on epidemics of HHD in hypertensive patients are still scarce. Díez found that hypertension increased the age-adjusted and risk-factor adjusted hazard of heart failure 2-fold in men and 3-fold in women [22]. However, heart failure and HHD are two distinct concepts. Ischemic heart disease, HHD, and aortic valve disease may all cause heart failure. Herweg et al. found that hypertension and HHD in patients are associated with pulmonary vein dilation but did not assess the epidemics of HHD in hypertensive patients [23]. Ekström et al. studied the transition from hypertension to HHD and heart failure in their PREFERS study [24]. However, the detailed correlation between HHD and hypertension symptoms is still unclear. Therefore, evaluation of both the HHD prevalence and the correlations between HHD and hypertension symptoms is in an urgent need, which may help establish a possibility that we could preidentify those hypertensive patients who are predisposed to developing HHD before the appearance of clinical manifestations and treat them with some preventive measures. To this end, we statistically analyzed the frequency of HHD and the correlations between HHD and selected symptoms in this study.

## MATERIALS AND METHODS

### Patients

All the 800 patients were randomly recruited from Qingdao, a metropolis in northeast China with a population of over 9 million people. Clinical data were collected at the Qingdao Fifth People’s Hospital, the Shandong Qingdao Hospital of Integrated Traditional and Western Medicine, and their local community health centers and township central hospitals. All the patients were diagnosed with hypertension according to “Hypertension Practice Guidelines in China”. The patients were selected with the random gender, marriage, and level of educations. Patients’ age ranged from 40 to 80 years old. Patients’ hypertension history age ranged from 1 to 20 years with or without family medical history of hypertension. The exclusion criteria included poor compliance, severe medical condition, and pregnancy or breast-feeding.

### Study design

All the patients were required to answer a questionnaire including medical history, family medical history, and lifestyle. The hypertension and its symptoms were diagnosed and assessed per ACC guidelines [25]. Cross-tabulation analysis was used to study the correlation between psychiatric indexes (annoy, amnesia, irritableness, depression, anxiety, and fear) and palpitation, the correlation between physical disorders (backache, lumbar debility, and numbness of limbs) and palpitation, and the correlation between high blood pressure symptoms (dizziness, daze, headache, and tinnitus) and palpitation presented in hypertensive patients. The blood routine examination was conducted for each patient.

### Statistical analysis

Categorical data were compared using the Chi-square test. Continuous data were expressed as mean standard deviation and compared using the Student’s t test (IBM SPSS Statistics 23). All *p*-values were two-tailed with the level of statistical significance set at 0.05.

## RESULTS

### The frequencies of patients suffering complicated cardiac disorders in hypertensive cohort

**Figure 1:**
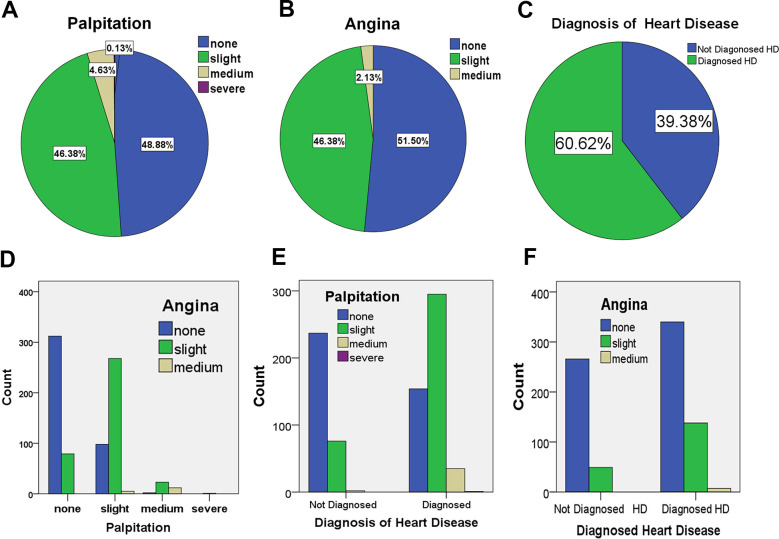
**The percentages of patients with cardiac disorders in hypertensive cohort.** The data was collected and analyzed from 800 cases of hypertension in northern China (n = 800). (**A**). The percentages of patients with different levels of palpitation in all patients with hypertension. (**B**). The percentages of patients with different levels of angina in all patients with hypertension. (**C**). The percentages of patients with diagnosis of heart disease in all patients with hypertension. (**D**). The analysis of cross-classification of palpitation and angina pectoris as corelated symptoms in patients with hypertension. (**E**). The analysis of cross-classification of palpitation and diagnosis of heart disease. (**F**). The analysis of cross-classification of Angina and diagnosis of heart disease.

Eight hundred patients with hypertension were randomly selected and surveyed. We found that 46.38% of patients had slight palpitation, 4.63% of patients had medium palpitation, and 0.13% of patients had severe palpitation (Figure 1A and Supplementary Table 1). We found that 46.38% of patients had slight angina pectoris, 2.13% of patients had medium-level angina, and no patients had severe angina (Figure 1B and Supplementary Table 2). More importantly, the diagnosis of heart disease demonstrates that a high frequency of heart disease among all hypertensive subjects as 60.62% (Figure 1C). To analyze the possible correlation of diagnosed heart disease, palpitation and angina, the cross-tabulation was performed. The significant correlation was observed between patients with palpitation and patients with angina (Supplementary Table 3). Most of patients with palpitation also have the symptom of angina (Figure 1D). Further, both of palpitation and angina are significantly correlated with the diagnosis of heart disease, especially palpitation presented dominantly in patients diagnosed with heart disease (Table 1A–1D and Figure 1E, 1F). The findings indicated that more than half of hypertensive patients in this study have complicating cardiac disease, and most of them shared both symptoms of palpitation and angina pectoris, which could be the associative indexes for diagnosis of HHD in hypertensive patients.

**Table 1A:** The analysis of cross-classification by diagnosis of heart disease * palpitation crosstabulation in patients with hypertension.

		**Palpitation**				**Total**
		**None**	**Slight**	**Medium**	**Severe**	
Diagnosed Heart Disease	Not Diagnosed HD	237	76	2	0	315
	Diagnosed HD	154	295	35	1	485
Total		391	371	37	1	800

**Table 1B:** Chi-square tests for diagnosis of heart disease * palpitation crosstabulation.

	**Value**	**df**	**Asymptotic significance (2-sided)**
Pearson Chi-Square	147.879a	3	.000
Likelihood Ratio	156.550	3	.000
Linear-by-Linear Association	140.304	1	.000
N of Valid Cases	800		

a. 2 cells (25.0%) have expected count less than 5. The minimum expected count is. 39.

**Table 1C:** The analysis of cross-classification by diagnosis of heart disease * angina crosstabulation in patients with hypertension.

		**Angina**			**Total**
		**None**	**Slight**	**Medium**	
Diagnosed Heart Disease	Not Diagnosed HD	266	49	0	315
	Diagnosed HD	340	138	7	485
Total		606	187	7	800

**Table 1D:** Chi-square tests for diagnosis of heart disease * angina crosstabulation.

	**Value**	**df**	**Asymptotic significance (2-sided)**
Pearson Chi-Square	23.323a	2	.000
Likelihood Ratio	26.484	2	.000
Linear-by-Linear Association	23.111	1	.000
N of Valid Cases	800		

a. 2 cells (33.3%) have expected count less than 5. The minimum expected count is 2.76.

### The psychiatric indexes correlate with palpitation presented in hypertensive patients

**Figure 2:**
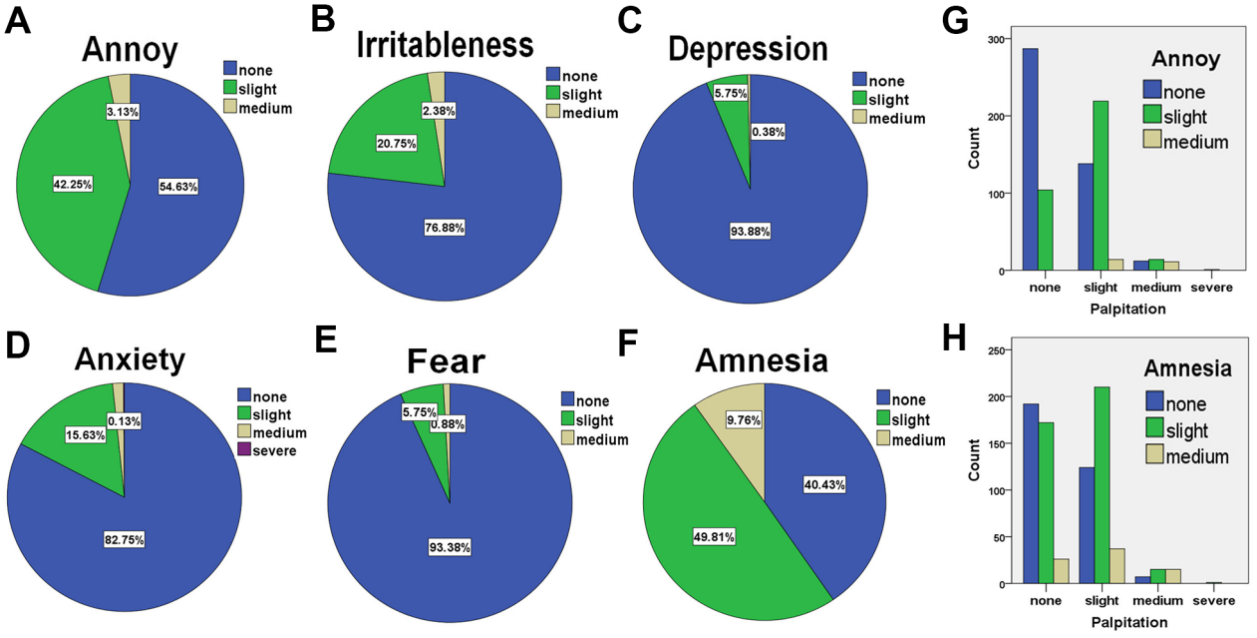
**The frequencies of hypertensive patients suffering various mental symptoms and their correlations with palpitation (n = 800).** (**A**) The percentages of patients who is annoyed with no reason. (**B**) The percentages of patients who are easy to get irritable. (**C**) The percentages of patients with depression. (**D**) The percentages of patients with anxiety. (**E**) The percentages of patients with fear. (**F**) The percentages of patients with amnesia. (**G**) The analysis of cross-classification of palpitation and psychiatric annoy in patients with hypertension. (**H**) The analysis of cross-classification of palpitation and psychiatric amnesia in patients with hypertension.

To identify the interrelated factors in HHD, we first checked if the mental disorders are correlated with palpitation in hypertensive patients. Different psychiatric indexes were checked from 800 cases randomly selected. We found the patients suffering annoy or amnesia approximately presented in half of the cases, patients suffering slight or medium mental annoy accounted for 45.38% in total, and patients with slight or medium amnesia accounted for 59.57% in total. However, the patients who have mental issues as either irritableness, depression, anxiety, or fear accounted for less than 25% of patients (Figure 2A – 2F and Supplementary Table 4A–4F). The high incidence rates of annoy and amnesia drove us to check if any correlations existing between these 2 mental symptoms and cardiac palpitation. Cross-tabulation was performed for patients with palpitation and patients with annoy or amnesia. It was found that patients with palpitation are significantly associated with patients with annoy (Figure 2G and Supplementary Table 5A, 5B). 25 cases out of a total 37 cases with medium palpitation also presented the symptom of annoy (67.6%) and 233 cases out of a total 371 cases with slight palpitation also presented the symptom of annoy (62.8%). This was similar for amnesia, the palpitation is significantly associated with amnesia in all patients (Figure 2H and Supplementary Table 5C, 5D). 30 cases out of a total 37 cases with medium palpitation also presented the amnesia (81.1%), and 247 cases out of a total 371 cases with slight palpitation also presented either slight or medium amnesia (66.6%). All the data suggested a positive correlation exists between palpitation and annoy/amnesia presented in hypertensive patients.

### The physical disorders correlate with palpitation presented in hypertensive patients

**Figure 3:**
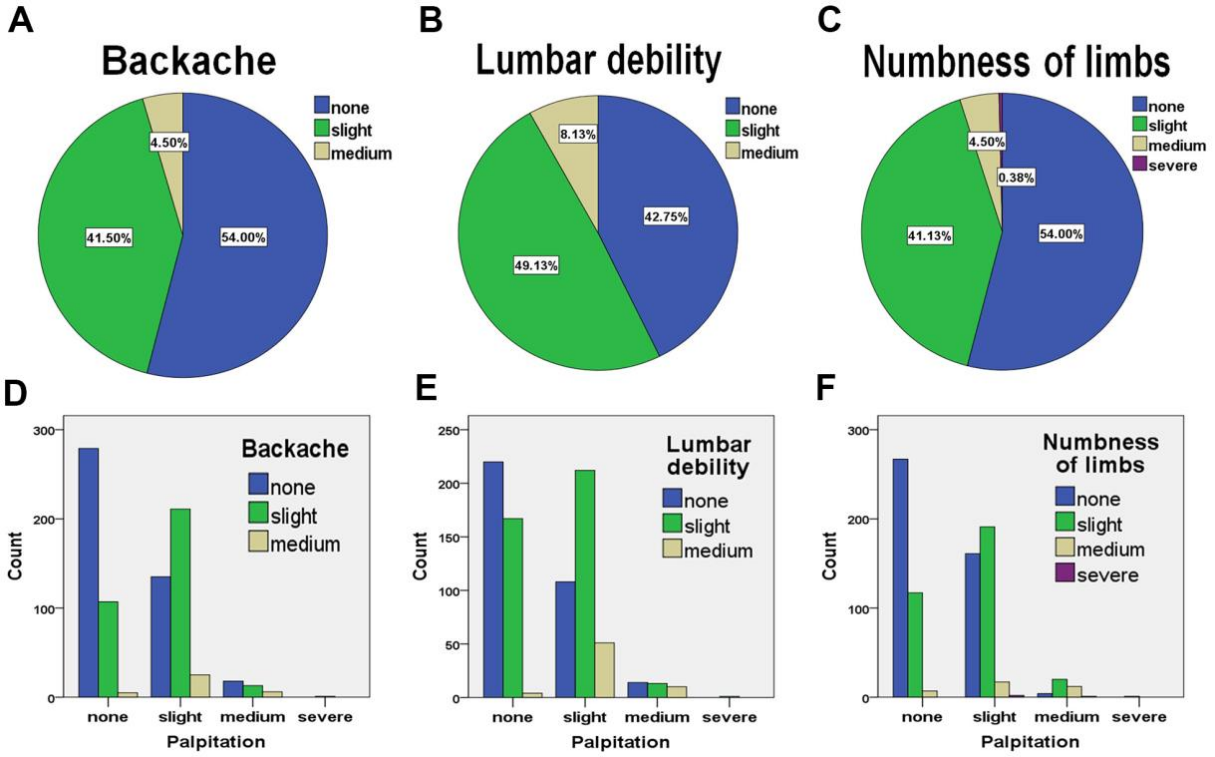
**The frequencies of hypertensive patients suffering various physical symptoms and their correlations with palpitation (n = 800).** The percentages of patients suffering backache (**A**), lumbar debility (**B**), and numbness of limbs (**C**). The analysis of cross-classification of palpitation and backache (**D**), palpitation and lumbar debility (**E**), and palpitation and numbness of limbs (**F**) in patients with hypertension.

Roughly 46% - 89.2% of patients suffered physical discomforts. In particular, patients suffering slight or medium backache accounted for 46% in total; patients suffering slight or medium lumbar debility 57.26% in total; and patients suffering numbness of limbs 46% in total (Figure 3A–3C and Supplementary Table 6A–6C). To assess the correlation between these physical disorders and palpitation, we performed cross-tabulation for patients with palpitation and patients with backache, lumbar debility or numbness of limbs. It was found that patients with palpitation were significantly correlated with patients with either backache and lumbar debility, or numbness of limbs (Figure 3D, 3F and Supplementary Table 7A–7F). A total of 236 cases out of 371 cases with slight palpitation suffered the symptoms of backache (63.6%); a total of 263 cases out of the 371 cases suffered the symptoms of lumbar debility (70.9%); a total of 210 cases out of the 371 cases suffered the symptoms of numbness of limbs (56.6%). A total of 23 cases out of 37 cases with medium palpitation suffered the symptoms of lumbar debility (62.2%); a total of 33 cases out of 37 cases suffered the symptoms of numbness of limbs (89.2%). Only one patient with severe palpitation suffered all the symptoms (back pain, lumbar debility and numbness of limbs). These results implied that the hypertensive patients with combined palpitation are susceptible to body disorders. This evidence is helpful for primary screen over cardiac complications in hypertension cohort.

### The symptoms of dizziness, being dazed, headache and tinnitus are correlated with palpitation presented in hypertensive patients

**Figure 4:**
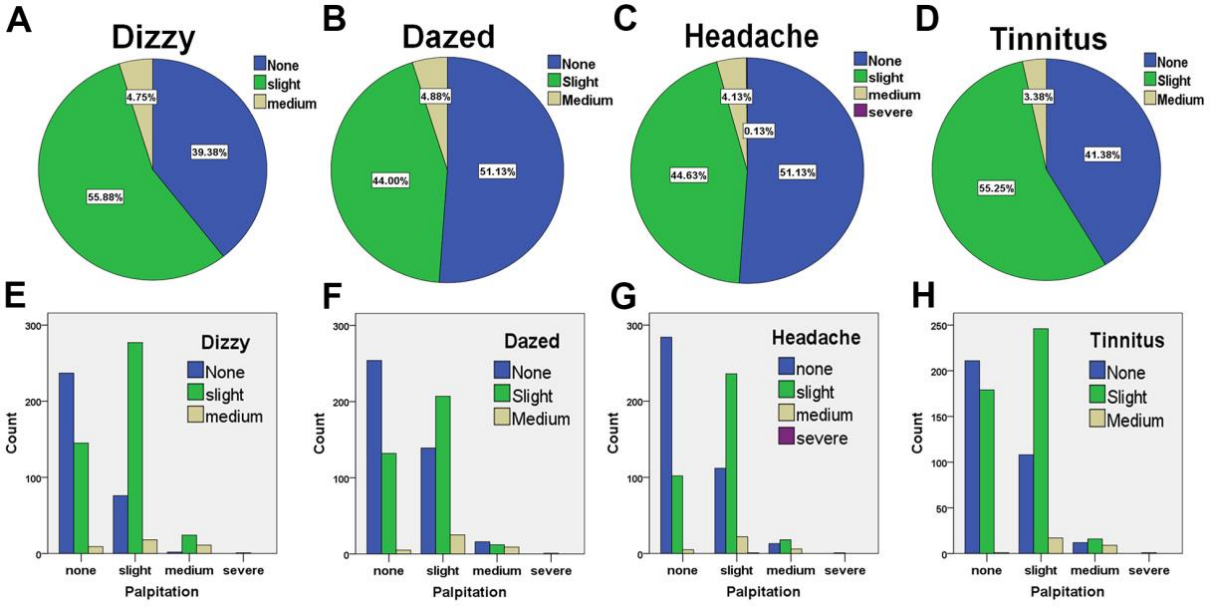
**The frequencies of hypertensive patients suffering cephalic symptoms and their correlations with palpitation (n = 800).** The percentages of patients suffering dizziness (**A**), daze (**B**), headache (**C**), and tinnitus (**D**). The analysis of cross-classification of palpitation and dizziness (**E**), palpitation and daze (**F**), palpitation and headache (**G**), and palpitation and tinnitus in patients with hypertension (**H**).

Eight hundred hypertensive patients suffered dizziness, dazed headache, or tinnitus. Among them, 60.7% of patients suffered occasionally slight or medium dizziness, 48.9% occasionally slight or intermediate daze, 48.8% headache, and 58.7% tinnitus (Figure 4A–4D and Supplementary Table 8A–8D). To assess the correlations between these symptoms and palpitation, we performed cross-tabulation for patients suffering palpitation and symptoms of dizziness, daze, headache, and tinnitus. It was found that palpitation is significantly correlated with symptoms of dizziness, daze, headache, or tinnitus (Figure 4E–4H and Supplementary Table 9A–9H). Among the 371 cases with slight palpitation, 295 cases (79.5%) suffered dizziness; 232 cases daze (62.5%), 258 cases (69.5%) headache, 263 cases (70.9%) tinnitus. Among the 37 cases with medium palpitation, 35 cases (94.6%) suffered dizziness, 21 cases (56.8%) daze, 24 (64.9%) cases headache, and 25 cases (67.6%) tinnitus. These results suggested that dizziness, daze, headache, and tinnitus could be the signs of complicated heart disease in hypertensive patients.

### The correlations between palpitation and blood routine indexes in hypertensive patients

**Figure 5:**
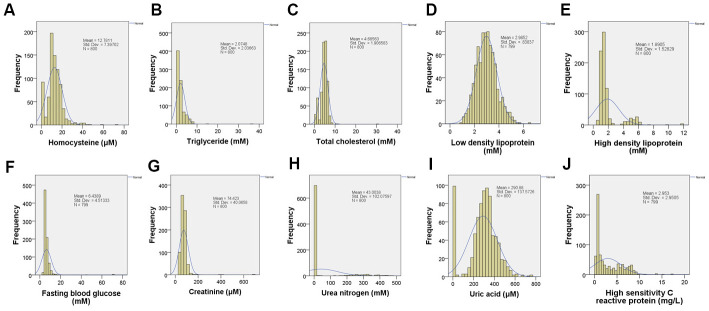
**The examination of blood routine.** The blood samples were collected and analyzed from hypertensive patients (n = 800). (**A**). Homocysteine; (**B**). Triglyceride; (**C**). Total cholesterol; (**D**). Low density lipoprotein; (**E**) High density lipoprotein; (**F**). Fasting blood glucose; (**G**). Creatinine; (**H**). Urea nitrogen; (**I**). Uric acid; (**J**). High sensitivity C reactive protein.

Blood routine lab is important for its accessibility and informativeness in hypertension management. We analyzed the 10 common indexes in blood routine from randomly selected 800 patients with hypertension (Supplementary Table 10 and Figure 5A–5J) and statistically checked their correlations with cardiac palpitation by SPSS Statistics 23. We found there were significantly positive correlations between palpitation and either homocysteine, total cholesterol, creatinine or uric acid (Supplementary Table 11A–11D), and the significantly negative correlations between palpitation and either triglyceride or high-density lipoprotein (Supplementary Table 11E, 11F).

## DISCUSSION

HHD refers to a group of disorders that includes heart failure, ischemic heart disease, and LVH. It is known that hypertension is a strong risk factor for heart disease, and HHD is the first cause of death associated with high blood pressure. Lethal ventricular arrhythmias and sudden cardiac death are more common in hypertensive patients than other types of heart diseases [16]. To initiate interventions as early as possible to prevent HHD from happening in hypertensive patients, it is necessary to assess the correlations between hypertension symptoms and HHD symptoms. To that end, we pooled the data from 800 hypertensive patients to systemically analyzed the correlation between the indexes of physical disorders, mental disorders, as well as lab routine blood indexes and heart disease symptoms. Patients in our study are more female than male as 61.25% versus 38.75% and have hypertension history from 1 year to 20 years. 21.3% of patients had family hypertension history (Supplementary Figure 1; Supplementary Tables 12, 13). Approximately, half of patients, who were diagnosed as heart disease (Supplementary Figure 2), presented cardiac symptoms as palpitation or angina pectoris, more patients were at slight level and a few patients at medium level, which indicated a high incidence of complicated heart diseases. Our results are consistent with previous study. Weber et al. mentioned in his article that hypertension contributes importantly to increased risk of major cardiovascular events [14]. Jeremy Slivnick et al. believed that high blood pressure could lead to severe heart diseases such as LVH, which incitingly leads to diastolic dysfunction [26].

Regarding the epidemics of HHD, Lamprea-Montealegre et al. studied the prevalence of hypertension in the U.S., but not the prevalence of HHD in hypertensive patients. In addition, they only estimated the associated cardiovascular disease without any diagnosis [27]. The investigation by Nkoke et al. in Cameroon focused on the predominance of HHD among the allover patients with cardiac diseases but not in the basis of hypertensive patients [28]. To our knowledge, all of the previous studies focused on the prevalence of hypertension, but not on HHD [29]. In contrast, the current study first time systemically investigated the frequencies of HHD among a large population of hypertensive patients. Our data will provide insight into the prevention and intervention against HHD.

The hypertension is estimated to affect more than 600 million people worldwide [14], who are living with a high risk of heart disease. However, due to the asymptomatic or subclinical symptoms in early stage of HHD, many patients had already been an advanced HHD when they first presented to their primary care providers. This dramatically increases the damages caused by HHD such as asymptomatic LVH or LVH subclinical symptoms. Although electrocardiogram and echocardiogram are readily available to diagnose LVH, it will not be anticipated for the primary care providers to order those examinations for their patients without notable cardiac symptoms [16]. As the result, the late diagnoses always predispose those patients to a worse situation.

In this study, we considered the presence of the complex symptoms of patients including physical disorders and metal disorders instead of looking at only cardiac disorders. It’s very interesting, the correlations between cardiac palpitation and mental disorders (annoy and amnesia) were observed. Actually, the link between heart disease and brain health has been made aware in recent years. It was observed that the abnormal blood flow might impair thinking ability. Chauvet-Gelinier and Bonin found that cardiac disorders impacted individual’s mental health [30]. Our study further assessed different indexes of mental conditions and found that only annoy and amnesia were significantly correlated to palpitation. Also, we found a close relationship between palpitation and some somatic disorders, such as backache, lumbar debility, numbness of limbs, and headache. Our results are consistent with previous study in this regard [31].

It is well known that age is the paramount risk factor for HHD. In this study, we took account of age by dividing patients into 4 groups of age, as years of 40-49, 50-59, 60-69 and more than 69. We found palpitation, which was considered as a representative clinical manifestation of heart disease, was significantly correlated with ages, but no significance was observed between angina and ages (Supplementary Table 14A–14D). These data demonstrate that age is a critical risk factor of HHD. Further, numbness of limbs, lumbar debility, backache, dazed head, headache, and tinnitus presented significance in correlation with ages. In contrast, amnesia, annoy, dizzy did not show significance in correlation with ages (Supplementary Table 14E–14V).

In summary, significant correlation exists between palpitation and angina. Significant correlation exists between palpitation and annoy / amnesia. Significant correlation exists between palpitation and backache / lumbar debility / numbness of limbs; and significant correlation exists between palpitation and dizziness / daze / headache / tinnitus. In addition, palpitation, numbness of limbs, lumbar debility, backache, dazed head, headache, and tinnitus were significantly correlated with age. These results provide clinical insights into early management of the modifiable antecedent clinical conditions. The success in management of these conditions will decrease HHD prevalence in elderly.

## Supplementary Material

Supplementary Figures

Supplementary Tables
