# Supplementary material for: The study of antecedent clinical manifestations of hypertensive heart disease in cohort of hypertension
Source: Aging (Albany NY). 2023 Feb 19;15(6):1890–917. doi: 10.18632/aging.204510 (PMC10085586; doi:10.18632/aging.204510)
Supplement: Supplementary Figures [file aging-15-204510-s001.pdf]

SUPPLEMENTARY FIGURES

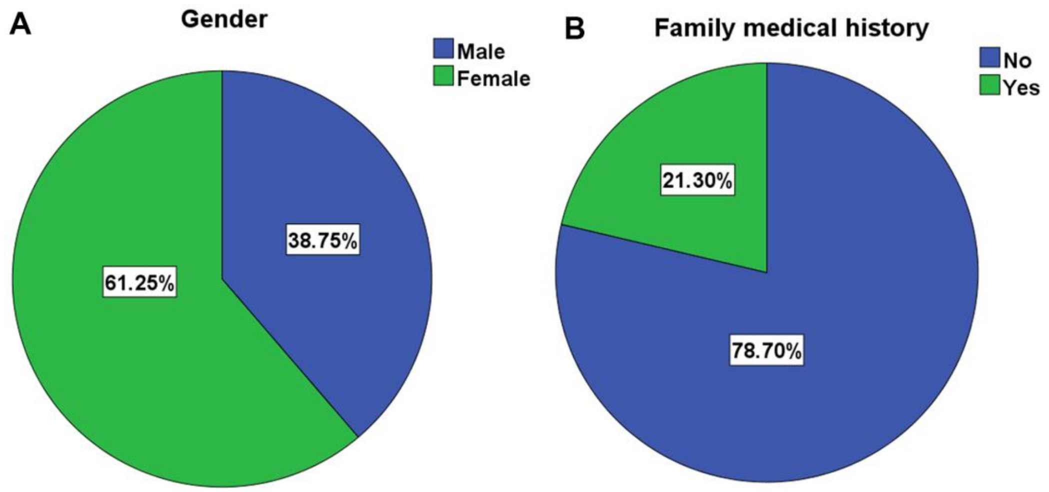

Supplementary Figure 1. The distribution of hypertensive patients with different genders and family medical history. (**A**) The frequencies of male or female patients in total hypertensive patients. (**B**) The frequencies of patients with or without familial inheritance of hypertension.

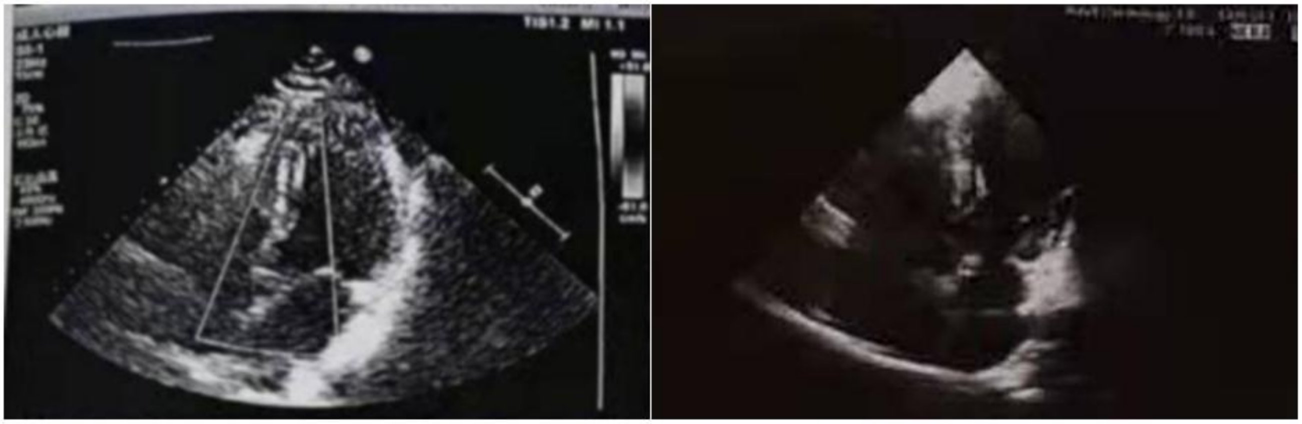

Supplementary Figure 2. The representative echocardiogram images from hypertensive patients. Left: diagnosis as no obvious abnormality of cardiac structure was found. Right: diagnosis as Pulmonary regurgitation (mild); Aortic regurgitation (mild); Left ventricular diastolic dysfunction; Extended aortic and decreased aortic elasticity.
