# Supplementary material for: The study of antecedent clinical manifestations of hypertensive heart disease in cohort of hypertension
Source: Aging (Albany NY). 2023 Feb 19;15(6):1890–917. doi: 10.18632/aging.204510 (PMC10085586; doi:10.18632/aging.204510)
Supplement: Supplementary Tables [file aging-15-204510-s002.pdf]

SUPPLEMENTARY TABLES

Supplementary Table 1. The percentage of patients with palpitation.

		Frequency	Percent	Valid percent	Cumulative percent
Valid	**none**	391	48.9	48.9	48.9
	**slight**	371	46.4	46.4	95.3
	**medium**	37	4.6	4.6	99.9
	**severe**	1	.1	.1	100.0
	Total	800	100.0	100.0	

Note: "none" indicates no palpitation ever occurred with the patient; "slight" indicates less than 3 times palpitation occurred per day with the patient; "medium" indicates around 3-12 times palpitation occurred per day with the patient which doesn't delay patient's routine work; "severe" indicates palpitation occurred more than 12 times per day.

Supplementary Table 2. The percentage of patients with angina.

		Frequency	Percent	Valid percent	Cumulative percent
Valid	**none**	412	51.5	51.5	51.5
	**slight**	371	46.4	46.4	97.9
	**medium**	17	2.1	2.1	100.0
	Total	800	100.0	100.0	

Note: "none" indicates no angina ever occurred with the patient; "slight" indicates less than 3 times angina occurred per day with the patient; "medium" indicates around 3-12 times angina occurred per day with the patient which doesn't delay patient's routine work; "severe" indicates angina occurred more than 12 times per day with undermined living quality on patient.

Supplementary Table 3A. The analysis of cross-classification by palpitation * angina crosstabulation.

		Angina			Total
		None	Slight	Medium	
**Palpitation**	none	312	79	0	391
	slight	98	268	5	371
	medium	2	23	12	37
	severe	0	1	0	1
Total		412	371	17	800

Supplementary Table 3B. Chi-square tests for palpitation * angina crosstabulation.

	Value	df	Asymptotic significance (2-sided)
Pearson Chi-Square	405.077[a]	6	.000
Likelihood Ratio	315.512	6	.000
Linear-by-Linear Association	270.656	1	.000
N of Valid Cases	800		

[a]4 cells (33.3%) have expected count less than 5. The minimum expected count is .02.

Supplementary Table 4A. The frequencies of patients who is annoyed with no reason.

		Frequency	Percent	Valid percent	Cumulative percent
Valid	none	437	54.6	54.6	54.6
	slight	338	42.3	42.3	96.9
	medium	25	3.1	3.1	100.0
	Total	800	100.0	100.0	

Supplementary Table 4B. The frequencies of patients with tendency to be irritable.

		Frequency	Percent	Valid percent	Cumulative percent
Valid	none	615	76.9	76.9	76.9
	slight	166	20.8	20.8	97.6
	medium	19	2.4	2.4	100.0
	Total	800	100.0	100.0	

Supplementary Table 4C. The frequencies of patients with depression.

		Frequency	Percent	Valid percent	Cumulative percent
Valid	none	751	93.9	93.9	93.9
	slight	46	5.8	5.8	99.6
	medium	3	.4	.4	100.0
	Total	800	100.0	100.0	

Supplementary Table 4D. The frequencies of patients with anxiety.

		Frequency	Percent	Valid percent	Cumulative percent
Valid	none	662	82.8	82.8	82.8
	slight	125	15.6	15.6	98.4
	medium	12	1.5	1.5	99.9
	severe	1	.1	.1	100.0
	Total	800	100.0	100.0	

Supplementary Table 4E. The frequencies of patients with fear.

		Frequency	Percent	Valid percent	Cumulative percent
Valid	none	747	93.4	93.4	93.4
	slight	46	5.8	5.8	99.1
	medium	7	.9	.9	100.0
	Total	800	100.0	100.0	

Supplementary Table 4F. The frequencies of patients with amnesia.

		Frequency	Percent	Valid percent	Cumulative percent
Valid	none	323	40.4	40.4	40.4
	slight	398	49.8	49.8	90.2
	medium	78	9.8	9.8	100.0
	Total	799	99.9	100.0	
Missing	System	1	.1		
Total		800	100.0		

Supplementary Table 5A. The analysis of cross-classification of palpitation and annoyed mentality in patients with hypertension.

		Annoy			Total
		None	Slight	Medium	
**Palpitation**	none	287	104	0	391
	slight	138	219	14	371
	medium	12	14	11	37
	severe	0	1	0	1
Total		437	338	25	800

Supplementary Table 5B. Chi-square tests for analysis of cross-classification of palpitation and annoyed mentality in patients with hypertension.

	Value	df	Asymptotic significance (2-sided)
Pearson Chi-Square	194.656[a]	6	.000
Likelihood Ratio	154.725	6	.000
Linear-by-Linear Association	126.564	1	.000
N of Valid Cases	800		

[a]4 cells (33.3%) have expected count less than 5. The minimum expected count is .03.

Supplementary Table 5C. The analysis of cross-classification by palpitation * amnesia crosstabulation.

		Amnesia			Total
		None	Slight	Medium	
**Palpitation**	none	192	172	26	390
	slight	124	210	37	371
	medium	7	15	15	37
	severe	0	1	0	1
Total		323	398	78	799

Supplementary Table 5D. Chi-square tests for palpitation * amnesia crosstabulation.

	Value	df	Asymptotic significance (2-sided)
Pearson Chi-Square	63.596[a]	6	.000
Likelihood Ratio	49.347	6	.000
Linear-by-Linear Association	38.971	1	.000
N of Valid Cases	799		

[a]4 cells (33.3%) have expected count less than 5. The minimum expected count is .10.

Supplementary Table 6A. The frequencies of patients with backache.

		Frequency	Percent	Valid percent	Cumulative percent
Valid	none	432	54.0	54.0	54.0
	slight	332	41.5	41.5	95.5
	medium	36	4.5	4.5	100.0
	Total	800	100.0	100.0	

Supplementary Table 6B. The frequencies of patients with lumbar debility.

		Frequency	Percent	Valid percent	Cumulative percent
Valid	none	342	42.8	42.8	42.8
	slight	393	49.1	49.1	91.9
	medium	65	8.1	8.1	100.0
	Total	800	100.0	100.0	

Supplementary Table 6C. The frequencies of patients with numbness of limbs.

		Frequency	Percent	Valid percent	Cumulative percent
Valid	none	432	54.0	54.0	54.0
	slight	329	41.1	41.1	95.1
	medium	36	4.5	4.5	99.6
	severe	3	.4	.4	100.0
	Total	800	100.0	100.0	

Supplementary Table 7A. The analysis of cross-classification by palpitation * backache crosstabulation in patients with hypertension.

		Backache			Total
		None	Slight	Medium	
**Palpitation**	none	279	107	5	391
	slight	135	211	25	371
	medium	18	13	6	37
	severe	0	1	0	1
Total		432	332	36	800

Supplementary Table 7B. Chi-square tests for palpitation * backache crosstabulation.

	Value	df	Asymptotic significance (2-sided)
Pearson Chi-Square	109.472[a]	6	.000
Likelihood Ratio	109.468	6	.000
Linear-by-Linear Association	81.646	1	.000
N of Valid Cases	800		

[a]4 cells (33.3%) have expected count less than 5. The minimum expected count is .05.

Supplementary Table 7C. The analysis of cross-classification by palpitation * lumbar debility crosstabulation in patients with hypertension.

		Lumbar debility			Total
		None	Slight	Medium	
**Palpitation**	none	220	167	4	391
	slight	108	212	51	371
	medium	14	13	10	37
	severe	0	1	0	1
Total		342	393	65	800

Supplementary Table 7D. Chi-square tests for palpitation * lumbar debility crosstabulation.

	Value	df	Asymptotic significance (2-sided)
Pearson Chi-Square	98.674[a]	6	.000
Likelihood Ratio	105.634	6	.000
Linear-by-Linear Association	75.273	1	.000
N of Valid Cases	800		

[a]4 cells (33.3%) have expected count less than 5. The minimum expected count is .08.

Supplementary Table 7E. The analysis of cross-classification by palpitation * numbness of limbs crosstabulation in patients with hypertension.

		Numbness of limbs				Total
		None	Slight	Medium	Severe	
**Palpitation**	none	267	117	7	0	391
	slight	161	191	17	2	371
	medium	4	20	12	1	37
	severe	0	1	0	0	1
Total		432	329	36	3	800

Supplementary Table 7F. Chi-square tests for palpitation * numbness of limbs crosstabulation.

	Value	df	Asymptotic significance (2-sided)
Pearson Chi-Square	137.443[a]	9	.000
Likelihood Ratio	106.711	9	.000
Linear-by-Linear Association	96.307	1	.000
N of Valid Cases	800		

[a]8 cells (50.0%) have expected count less than 5. The minimum expected count is .00.

Supplementary Table 8A. The frequencies of patients with dizzy.

		Frequency	Percent	Valid percent	Cumulative percent
Valid	None	315	39.4	39.4	39.4
	slight	447	55.9	55.9	95.3
	medium	38	4.8	4.8	100.0
	Total	800	100.0	100.0	

Supplementary Table 8B. The frequencies of patients being dazed.

		Frequency	Percent	Valid percent	Cumulative percent
Valid	None	409	51.1	51.1	51.1
	Slight	352	44.0	44.0	95.1
	Medium	39	4.9	4.9	100.0
	Total	800	100.0	100.0	

Supplementary Table 8C. The frequencies of patients with headache.

		Frequency	Percent	Valid percent	Cumulative percent
Valid	none	409	51.1	51.1	51.1
	slight	357	44.6	44.6	95.8
	medium	33	4.1	4.1	99.9
	severe	1	.1	.1	100.0
	Total	800	100.0	100.0	

Supplementary Table 8D. The frequencies of patients with tinnitus.

		Frequency	Percent	Valid percent	Cumulative percent
Valid	None	331	41.4	41.4	41.4
	Slight	442	55.3	55.3	96.6
	Medium	27	3.4	3.4	100.0
	Total	800	100.0	100.0	

Supplementary Table 9A. The analysis of cross-classification by palpitation * dizzy crosstabulation in patients with hypertension.

		Dizzy			Total
		None	Slight	Medium	
**Palpitation**	none	237	145	9	391
	slight	76	277	18	371
	medium	2	24	11	37
	severe	0	1	0	1
Total		315	447	38	800

Supplementary Table 9B. Chi-square tests for palpitation * dizzy crosstabulation.

	Value	df	Asymptotic significance (2-sided)
Pearson Chi-Square	192.276[a]	6	.000
Likelihood Ratio	175.322	6	.000
Linear-by-Linear Association	148.627	1	.000
N of Valid Cases	800		

[a]4 cells (33.3%) have expected count less than 5. The minimum expected count is .05.

Supplementary Table 9C. The analysis of cross-classification by palpitation * dazed crosstabulation in patients with hypertension.

		Dazed			Total
		None	Slight	Medium	
Palpitation	none	254	132	5	391
	slight	139	207	25	371
	medium	16	12	9	37
	severe	0	1	0	1
Total		409	352	39	800

Supplementary Table 9D. Chi-square tests for palpitation * dazed crosstabulation.

	Value	df	Asymptotic significance (2-sided)
Pearson Chi-Square	93.803[a]	6	.000
Likelihood Ratio	84.350	6	.000
Linear-by-Linear Association	65.320	1	.000
N of Valid Cases	800		

[a]4 cells (33.3%) have expected count less than 5. The minimum expected count is .05.

Supplementary Table 9E. The analysis of cross-classification by palpitation * headache crosstabulation in patients with hypertension.

		Headache				Total
		None	Slight	Medium	Severe	
Palpitation	none	284	102	5	0	391
	slight	112	236	22	1	371
	medium	13	18	6	0	37
	severe	0	1	0	0	1
Total		409	357	33	1	800

Supplementary Table 9F. Chi-square tests for palpitation * headache crosstabulation.

	Value	df	Asymptotic significance (2-sided)
Pearson Chi-Square	155.376[a]	9	.000
Likelihood Ratio	156.455	9	.000
Linear-by-Linear Association	122.742	1	.000
N of Valid Cases	800		

[a]8 cells (50.0%) have expected count less than 5. The minimum expected count is .00.

Supplementary Table 9G. The analysis of cross-classification by palpitation * tinnitus crosstabulation in patients with hypertension.

		Tinnitus			Total
		None	Slight	Medium	
**Palpitation**	none	211	179	1	391
	slight	108	246	17	371
	medium	12	16	9	37
	severe	0	1	0	1
Total		331	442	27	800

Supplementary Table 9H. Chi-square tests for palpitation * tinnitus crosstabulation.

	Value	df	Asymptotic significance (2-sided)
Pearson Chi-Square	106.500[a]	6	.000
Likelihood Ratio	86.896	6	.000
Linear-by-Linear Association	63.782	1	.000
N of Valid Cases	800		

[a]4 cells (33.3%) have expected count less than 5. The minimum expected count is .03.

Supplementary Table 10. Descriptive statistics of blood routine index.

	N	Minimum	Maximum	Mean	Std. deviation
Homocysteine (μM)	800	.65	72.80	12.7811	7.39702
Triglyceride (mM)	800	.00	36.00	2.0748	2.03663
Total cholesterol (mM)	800	.42	30.93	4.68563	1.906583
Low density lipoprotein (mM)	799	.98	6.55	2.9852	.83837
High density lipoprotein (mM)	800	.52	11.68	1.8905	1.52829
Fasting blood glucose (mM)	799	2.68	70.19	6.4389	4.51333
Creatinine (μM)	800	30.0	694.2	74.423	40.0658
Urea nitrogen (mM)	800	2.16	469.00	43.0038	102.07597
Uric acid (μM)	800	3.5	766.3	290.880	137.5726
High sensitivity C reactive protein (mg/L)	799	.2	17.3	2.953	2.9505
Valid N (listwise)	797				

Supplementary Table 11A. Correlations of grade of palpitation and homocysteine value in patients with hypertension.

			Palpitation	Homocysteine
Spearman's rho	Palpitation	Correlation Coefficient	1.000	.257**
		Sig. (2-tailed)	.	.000
		N	800	800
	Homocysteine	Correlation Coefficient	.257**	1.000
		Sig. (2-tailed)	.000	.
		N	800	800

**Correlation is significant at the 0.01 level (2-tailed).

Supplementary Table 11B. Correlations of grade of palpitation and total cholesterol value in patients with hypertension correlations.

			Palpitation	Total cholesterol
Spearman's rho	Palpitation	Correlation Coefficient	1.000	.178**
		Sig. (2-tailed)	.	.000
		N	800	800
	Total cholesterol	Correlation Coefficient	.178**	1.000
		Sig. (2-tailed)	.000	.
		N	800	800

**Correlation is significant at the 0.01 level (2-tailed).

Supplementary Table 11C. Correlations of grade of palpitation and creatinine value in patients with hypertension correlations.

			Palpitation	Creatinine
Spearman's rho	Palpitation	Correlation Coefficient	1.000	.087*
		Sig. (2-tailed)	.	.014
		N	800	800
	Creatinine	Correlation Coefficient	.087*	1.000
		Sig. (2-tailed)	.014	.
		N	800	800

*Correlation is significant at the 0.05 level (2-tailed).

Supplementary Table 11D. Correlations of grade of palpitation and uric acid value in patients with hypertension correlations.

			Palpitation	Uric acid
Spearman's rho	Palpitation	Correlation Coefficient	1.000	.203**
		Sig. (2-tailed)	.	.000
		N	800	800
	Uric acid	Correlation Coefficient	.203**	1.000
		Sig. (2-tailed)	.000	.
		N	800	800

**Correlation is significant at the 0.01 level (2-tailed).

Supplementary Table 11E. Correlations of grade of palpitation and triglyceride value in patients with hypertension correlations.

			Palpitation	Triglyceride
Spearman's rho	Palpitation	Correlation Coefficient	1.000	-.224**
		Sig. (2-tailed)	.	.000
		N	800	800
	Triglyceride	Correlation Coefficient	-.224**	1.000
		Sig. (2-tailed)	.000	.
		N	800	800

**Correlation is significant at the 0.01 level (2-tailed).

Supplementary Table 11F. Correlations of grade of palpitation and high-density lipoprotein value in patients with hypertension Correlations.

			Palpitation	High density lipoprotein
Spearman's rho	Palpitation	Correlation Coefficient	1.000	-.156**
		Sig. (2-tailed)	.	.000
		N	800	800
	High density lipoprotein	Correlation Coefficient	-.156**	1.000
		Sig. (2-tailed)	.000	.
		N	800	800

**Correlation is significant at the 0.01 level (2-tailed).

Supplementary Table 12. Gender.

		Frequency	Percent	Valid percent	Cumulative percent
Valid	Male	310	38.8	38.8	38.8
	Female	490	61.3	61.3	100.0
	Total	800	100.0	100.0	

Supplementary Table 13. Family medical history.

		Frequency	Percent	Valid percent	Cumulative percent
Valid	No	628	78.5	78.7	78.7
	Yes	170	21.3	21.3	100.0
	Total	798	99.8	100.0	
Missing	System	2	.3		
Total		800	100.0		

Supplementary Table 14A. The analysis of cross-classification by palpitation * Age crosstabulation in patients with hypertension.

		Palpitation				Total
		None	Slight	Medium	Severe	
Age Grades	40-49	10	6	0	0	16
	50-59	5	0	0	0	5
	60-69	165	135	6	0	306
	>69	211	230	31	1	473
Total		391	371	37	1	800

Supplementary Table 14B. Chi-square tests for palpitation * age crosstabulation.

	Value	df	Asymptotic significance (2-sided)
Pearson Chi-Square	20.245[a]	9	.016
Likelihood Ratio	24.106	9	.004
Linear-by-Linear Association	14.290	1	.000
N of Valid Cases	800		

[a]8 cells (50.0%) have expected count less than 5. The minimum expected count is .01.

Supplementary Table 14C. The analysis of cross-classification by angina * age crosstabulation in patients with hypertension.

		Angina			Total
		None	Slight	Medium	
Age Grades	40-49	12	4	0	16
	50-59	4	1	0	5
	60-69	238	63	5	306
	>69	352	119	2	473
Total		606	187	7	800

Supplementary Table 14D. Chi-square tests for angina * age crosstabulation.

	Value	df	Asymptotic significance (2-sided)
Pearson Chi-Square	5.296[a]	6	.506
Likelihood Ratio	5.356	6	.499
Linear-by-Linear Association	.317	1	.574
N of Valid Cases	800		

[a]7 cells (58.3%) have expected count less than 5. The minimum expected count is .04.

Supplementary Table 14E. The analysis of cross-classification by amnesia * age crosstabulation in patients with hypertension.

		Amnesia			Total
		None	Slight	Medium	
Age Grades	40-49	5	9	2	16
	50-59	4	1	0	5
	60-69	129	153	24	306
	>69	185	235	52	472
Total		323	398	78	799

Supplementary Table 14F. Chi-square tests for amnesia * age crosstabulation.

	Value	df	Asymptotic significance (2-sided)
Pearson Chi-Square	6.228[a]	6	.398
Likelihood Ratio	6.611	6	.358
Linear-by-Linear Association	.868	1	.351
N of Valid Cases	799		

[a]4 cells (33.3%) have expected count less than 5. The minimum expected count is .49.

Supplementary Table 14G. The analysis of cross-classification by annoy * age crosstabulation in patients with hypertension.

		Annoy			Total
		None	Slight	Medium	
Age Grades	40-49	12	3	1	16
	50-59	4	1	0	5
	60-69	167	131	8	306
	>69	254	203	16	473
Total		437	338	25	800

Supplementary Table 14H. Chi-square tests for annoy * age crosstabulation.

	Value	df	Asymptotic significance (2-sided)
Pearson Chi-Square	5.642[a]	6	.465
Likelihood Ratio	6.192	6	.402
Linear-by-Linear Association	1.652	1	.199
N of Valid Cases	800		

[a]4 cells (33.3%) have expected count less than 5. The minimum expected count is .16.

Supplementary Table 14I. The analysis of cross-classification by numbness of limbs * age crosstabulation in patients with hypertension.

		Numbness of limbs				Total
		None	Slight	Medium	Severe	
Age Grades	40-49	13	3	0	0	16
	50-59	3	2	0	0	5
	60-69	184	116	6	0	306
	>69	232	208	30	3	473
Total		432	329	36	3	800

Supplementary Table 14J. Chi-square tests for numbness of limbs * age crosstabulation.

	Value	df	Asymptotic significance (2-sided)
Pearson Chi-Square	21.136[a]	9	.012
Likelihood Ratio	24.133	9	.004
Linear-by-Linear Association	18.789	1	.000
N of Valid Cases	800		

[a]8 cells (50.0%) have expected count less than 5. The minimum expected count is .02.

Supplementary Table 14K. The analysis of cross-classification by lumbar debility * age crosstabulation in patients with hypertension.

		Debility of lumbar			Total
		None	Slight	Medium	
Age Grades	40-49	5	10	1	16
	50-59	3	2	0	5
	60-69	153	125	28	306
	>69	181	256	36	473
Total		342	393	65	800

Supplementary Table 14L. Chi-square tests for lumbar debility * age crosstabulation.

	Value	df	Asymptotic significance (2-sided)
Pearson Chi-Square	15.193[a]	6	.019
Likelihood Ratio	15.634	6	.016
Linear-by-Linear Association	2.429	1	.119
N of Valid Cases	800		

[a]4 cells (33.3%) have expected count less than 5. The minimum expected count is .41.

Supplementary Table 14M. The analysis of cross-classification by backache * age crosstabulation in patients with hypertension.

		Backache			Total
		None	Slight	Medium	
Age Grades	40-49	14	1	1	16
	50-59	1	4	0	5
	60-69	165	122	19	306
	>69	252	205	16	473
Total		432	332	36	800

Supplementary Table 14N. Chi-square tests for backache * age crosstabulation.

	Value	df	Asymptotic significance (2-sided)
Pearson Chi-Square	15.233[a]	6	.019
Likelihood Ratio	17.486	6	.008
Linear-by-Linear Association	.449	1	.503
N of Valid Cases	800		

[a]4 cells (33.3%) have expected count less than 5. The minimum expected count is .22.

Supplementary Table 14O. The analysis of cross-classification by dizzy * age crosstabulation in patients with hypertension.

		Dizzy			Total
		None	Slight	Medium	
Age Grades	40-49	9	6	1	16
	50-59	2	3	0	5
	60-69	138	158	10	306
	>69	166	280	27	473
Total		315	447	38	800

Supplementary Table 14P. Chi-square tests for dizzy * age crosstabulation.

	Value	df	Asymptotic significance (2-sided)
Pearson Chi-Square	11.447[a]	6	.076
Likelihood Ratio	11.765	6	.067
Linear-by-Linear Association	9.168	1	.002
N of Valid Cases	800		

[a]4 cells (33.3%) have expected count less than 5. The minimum expected count is .24.

Supplementary Table 14Q. The analysis of cross-classification by dazed * age crosstabulation in patients with hypertension.

		Dazed			Total
		None	Slight	Medium	
Age Grades	40-49	13	3	0	16
	50-59	3	2	0	5
	60-69	179	116	11	306
	>69	214	231	28	473
Total		409	352	39	800

Supplementary Table 14R. Chi-square tests for dazed * age crosstabulation.

	Value	df	Asymptotic significance (2-sided)
Pearson Chi-Square	19.914[a]	6	.003
Likelihood Ratio	21.204	6	.002
Linear-by-Linear Association	18.901	1	.000
N of Valid Cases	800		

[a]4 cells (33.3%) have expected count less than 5. The minimum expected count is .24.

Supplementary Table 14S. The analysis of cross-classification by headache * age crosstabulation in patients with hypertension.

		Headache				Total
		None	Slight	Medium	Severe	
Age Grades	40-49	12	3	1	0	16
	50-59	5	0	0	0	5
	60-69	169	131	5	1	306
	>69	223	223	27	0	473
Total		409	357	33	1	800

Supplementary Table 14T. Chi-square tests for headache * age crosstabulation.

	Value	df	Asymptotic significance (2-sided)
Pearson Chi-Square	21.597[a]	9	.010
Likelihood Ratio	25.329	9	.003
Linear-by-Linear Association	11.744	1	.001
N of Valid Cases	800		

[a]8 cells (50.0%) have expected count less than 5. The minimum expected count is .01.

Supplementary Table 14U. The analysis of cross-classification by Tinnitus * age crosstabulation in patients with hypertension.

		Tinnitus			Total
		None	Slight	Medium	
Age Grades	40-49	14	2	0	16
	50-59	3	2	0	5
	60-69	143	155	8	306
	>69	171	283	19	473
Total		331	442	27	800

Supplementary Table 14V. Chi-square tests for tinnitus * age crosstabulation.

	Value	df	Asymptotic significance (2-sided)
Pearson Chi-Square	24.168[a]	6	.000
Likelihood Ratio	25.325	6	.000
Linear-by-Linear Association	21.611	1	.000
N of Valid Cases	800		

[a]4 cells (33.3%) have expected count less than 5. The minimum expected count is .17.
